# Hydrophilic Species Are the Most Biodegradable Components of Freshwater Dissolved Organic Matter

**DOI:** 10.1021/acs.est.3c02175

**Published:** 2023-08-30

**Authors:** Charlotte Grasset, Marloes Groeneveld, Lars J. Tranvik, Luke P. Robertson, Jeffrey A. Hawkes

**Affiliations:** † Department of Ecology and Genetics, Limnology, Uppsala University, Uppsala 75236, Sweden; ‡ Department of Pharmaceutical Biosciences, Uppsala University, Uppsala 75237, Sweden; § Department of Chemistry, BMC, Uppsala University, Uppsala 75237, Sweden

**Keywords:** dissolved organic matter, biodegradability, mass spectrometry, electrospray ionization, freshwater

## Abstract

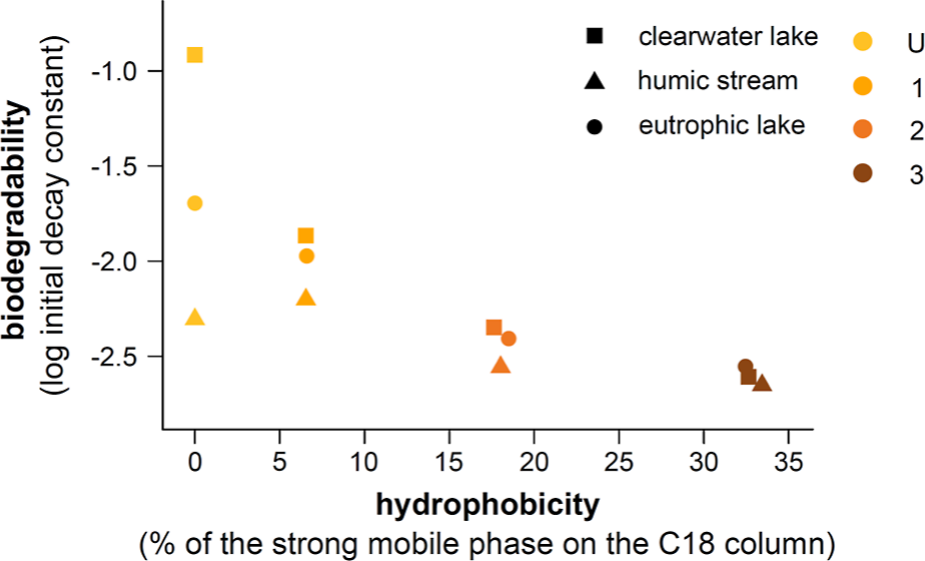

Aquatic dissolved organic matter (DOM) is a crucial component of the global carbon cycle, and the extent to which DOM escapes mineralization is important for the transport of organic carbon from the continents to the ocean. DOM persistence strongly depends on its molecular properties, but little is known about which specific properties cause the continuum in reactivity among different dissolved molecules. We investigated how DOM fractions, separated according to their hydrophobicity, differ in biodegradability across three different inland water systems. We found a strong negative relationship between hydrophobicity and biodegradability, consistent for the three systems. The most hydrophilic fraction was poorly recovered by solid-phase extraction (SPE) (3–28% DOC recovery) and was thus selectively missed by mass spectrometry analysis during SPE. The change in DOM composition after incubation was very low according to SPE–ESI (electrospray ionization)–mass spectrometry (14% change, while replicates had 11% change), revealing that this method is sub-optimal to assess DOM biodegradability, regardless of fraction hydrophobicity. Our results demonstrate that SPE–ESI mass spectrometry does not detect the most hydrophilic and most biodegradable species. Hence, they question our current understanding of the relationships between DOM biodegradability and its molecular composition, which is built on the use of this method.

## Abstract 2

Dissolved organic matter was separated based on hydrophobicity, and each fraction’s biological lability and composition were tested. We found that more hydrophilic fractions were more labile and more challenging to characterize.

## Introduction

Inland waters are a significant source of atmospheric greenhouse gases^1,2^ and they export substantial amounts of organic matter to the sea.^3,4^ Dissolved organic matter (DOM) is a major precursor for greenhouse gas emissions^5^ and the extent to which it escapes mineralization is important for the transport of organic carbon from the continents to the ocean. DOM biological degradation is the breakdown of DOM into smaller compounds via reactions that are mediated by microorganisms (i.e., biotic oxidation and hydrolysis). It is controlled by extrinsic factors (e.g., temperature, light^6^) and the intrinsic properties of DOM chemical constituents.^7−9^ Two theories explaining how the intrinsic chemical properties of DOM control DOM biodegradation or persistence are actively debated.^10,11^ According to the first theory, compounds may be difficult to degrade because of their chemical structure^11^ that makes them inherently stable. According to the second theory, termed “the dilution hypothesis”, compounds may be difficult to use for microbes because they are extremely diverse with slight structural variations, making each substrate of vanishingly low concentration.^10^ Evidence for either theory requires investigation of DOM biodegradability at a molecular level to examine the lability of individual compounds at varying concentrations and in different ecological contexts.

Since DOM is composed of countless compounds with different reactivities, the rate of decay of DOM in incubation studies is not exponential, as would be expected from a single substrate with one first-order reaction rate, but is instead built up of a continuum of different exponential decay rates.^12,13^ Previous work has shown that when bulk DOM is separated into low and high apparent molecular weight fractions with ultrafiltration (LMW and HMW, respectively), the HMW fraction is more biodegradable.^14,15^ This result has led to the so-called “size-reactivity continuum model”, in which DOM is theorized to be degraded to progressively smaller and less biodegradable forms over time.^16^ However, contradicting relationships between size and biodegradability have been found for both apparent^17,18^ and actual molecular weight.^19,20^ Experimental studies that relate other characteristics of bulk DOM than size to biodegradability are consequently needed.

The degree of hydrophobicity is another major characteristic that varies among DOM components, but this has not been previously related to bulk DOM biodegradability. The hydrophilic character of a molecule describes its affinity to water and is related to its polarity (i.e., referring to the spatial distribution of electron density within the molecule). DOM mixtures can be separated by polarity since more hydrophobic species retain better on a hydrophobic material such as C_18_-bonded silica or styrene/divinyl benzene (e.g., Agilent PPL). Many labile biomolecular classes (e.g., sugars, free amino acids, and peptides)^11,21^ are hydrophilic, but surprisingly, to our knowledge, no study has tested how DOM fractions of different hydrophobicity differ in biodegradability.

Recent research into DOM biodegradability has suggested that the presence of some constituents of DOM can promote (or suppress) the degradation of others,^22,23^ sometimes referred to as a “priming effect”. Therefore, the bulk biodegradability of DOM when all individual molecules occur together may not correspond to the sum of the biodegradability of individual molecules or fractions when incubated separately. Consequently, it is not clear if DOM fractions of different hydrophobicity would interact in ways that affect their biodegradability when they are degrading together.

An increasing number of studies have investigated the reactivity of DOM at the molecular level using high-resolution mass spectrometry (MS) techniques.^7,24−27^ Specifically, in inland waters, the exponential decays of a multitude of compounds composing DOM have been related to their characteristics (O/C, H/C, molecular weight) using MS.^19^ Problematically, in high-resolution MS analyses, the most hydrophilic fraction is excluded during the preliminary extraction and concentration step and during ionization.^28,29^ Therefore, the results of MS studies and other techniques that use extraction isolates [e.g., with solid-phase extraction (SPE)] or electrospray ionization (ESI) MS, are biased toward more hydrophobic DOM and may not represent the biodegradability of bulk DOM.

In this study, we hypothesized that the most hydrophilic fractions of DOM would be the most biodegradable. Accordingly, the most hydrophobic fractions would persist due to the low microbial ability to degrade them and their limited accessibility when dissolved in water (due to aggregation^30^). We used the loss of organic carbon (i.e., mineralization) as a measure of biodegradability and compared the biological DOC loss of four fractions of differing hydrophobicity separated from three contrasting inland water samples (humic stream, clearwater, and eutrophic lake samples). In addition, we monitored the DOC loss of all fractions pooled together and compared it to a theoretical DOC loss, assuming that the different fractions did not interact during degradation.

## Materials and Methods

### Sampling Sites

Three inland water sites of the Uppland region (Sweden), one humic stream, and two lakes with contrasting nutrient status and watershed characteristics were selected because of their expected differences in DOM quality (Table S1). Fiby is a humic stream and thus has a short water retention time and a high abundance of fresh terrestrially-derived DOM. Långsjön (“clearwater lake”; theoretical water residence time: 3–8 years) and Alstasjön (“eutrophic lake”; theoretical water residence time: 6 days) are mesoeutrophic and hypereutrophic lakes, respectively, and thus are expected to have higher contributions of in situ-produced DOM than the humic stream.

To obtain the chemical water characteristics of the sites, total phosphorus (TP), total nitrogen (TN), and pH were measured on 60 μm plankton net filtered samples, and DOC was measured on Whatman GF/F filtered water samples collected in October and November 2021 and stored at 4 °C in the dark before analysis. TP concentrations were measured colorimetrically with a UV–vis spectrophotometer (Lambda 40; PerkinElmer; Waltham, Massachusetts, USA) using the molybdenum-blue method.^31^ TN concentrations were determined on a total organic carbon (TOC)/TN analyzer (Shimadzu TOC-L/TNM-L, Kyoto, Japan). DOC concentrations were determined using a Sievers M9 TOC analyzer (GE Analytical Instruments, Boulder, Colorado, USA). pH was determined with a Metrohm 826 pH Mobile meter.

### Sample Concentration by Reverse Osmosis

About 50–150 L of water from each site was 3 to 22 times concentrated to a final concentration of approximately 140 mg DOC L^–1^ by reverse osmosis (Real Soft PROS/2S unit) in October and November 2021. Prior to concentration by reverse osmosis, the water was sequentially filtered through 5, 0.5, and 0.2 μm pore size membrane filters with a submersible pump through 10 in. filter cartridges and passed through a strongly acidic cation exchange resin (Dowex 50W X8, Dow Chemical Company). This concentration step was necessary to work with limited volumes of water during the sample fractionation prior to redissolution to reach a DOC concentration of around 10 mg L^–1^ in the incubation vials.

Additionally, a smaller water sample (ca. 1 L) was filtered through a 60 μm plankton net to remove large particles and serve as a microbial inoculum during the incubation. All water samples were kept at 4 °C in the dark before the fractionation or before the start of the incubation.

### Sample Fractionation

Water samples were filtered within 24 h before fractionation with pre-combusted GF/F filters. For best sample retention, methanol (MeOH, ∼50 mL) and trifluoroacetic acid (TFA, ∼1 mL) were added to approximately 1 L of concentrated lake water sample to bring each to 5% MeOH and 0.1% TFA. Two separate C18 fractionations were then performed to reduce the loading volume and improve the separation efficiency. Each sample was first individually loaded onto a preconditioned flash column (Biotage Sfär Duo C_18_, 120 g, 100 Å, 30 μm). After each sample was fully loaded onto the column, the unretained material was eluted with the manufacturer-listed dead volume (160 mL) of 5% MeOH (0.1% TFA). The entire unretained eluent (∼1 L) was collected into a bottle and labeled as the “unretained” fraction. Retained material was then eluted with 250 mL of 95% acetonitrile (CH_3_CN; 0.1% TFA) and collected into a second bottle (250 mL), which was labeled the “retained” fraction. Both the “unretained” and “retained” fractions were lyophilized. The “unretained” fraction was weighed and stored in the freezer. The “retained” fraction was then purified using preparative HPLC (Kinetex XB-C_18_, 150 × 21.2 mm, 100 Å, 5 μm) using a gradient consisting of isocratic 5% CH_3_CN (0.1% TFA) for 5 min, then to 95% CH_3_CN (0.1% TFA) over the next 50 min. The column was then eluted with 95% CH_3_CN (0.1% TFA) for 5 min. The flow rate was 9 mL/min, and fractions were collected every 60 s into pre-weighed glass test tubes. The test tubes were evaporated overnight using a centrifugal evaporator (30 °C) and weighed again to reveal the weight of each fraction (Figure S1).

### Incubation Preparation

Six different water samples were incubated for each site. Four fractions of increasing hydrophobicity were separated from concentrated DOC samples: fraction U (“unretained”) corresponds to the unretained, most hydrophilic fraction, and fractions 1 to 3 are the retained fractions of increasing hydrophobicity. C (“combined”) is the recombination of U, 1, 2, and 3 in their original abundances to recreate a sample that is close to the original sample. In addition, the original concentrated water sample (abbreviated O for “original”) was included in the incubations and compared to the recreated original sample in order to assess if the fractionation affected the biodegradability of the samples.

The weights of the test tubes obtained after fractionation were evaluated along with the gradient conditions to arbitrarily choose fractions 1, 2, and 3 with enough material in all three sites to make incubations with sufficient carbon concentration for analysis (Figure S1). Since most material was eluted within the first 35 min (corresponding to the first 35 tubes), the test tube ranges selected were tubes 1–11 (fraction 1), tubes 12–18 (fraction 2), and tubes 19–35 (fraction 3). Taking into account the dead volume of the column, fractions 1, 2, and 3 were eluted with 5–10, 10–22, and 22–53% CH_3_CN, respectively.

Fractions U, 1, 2, and 3 were then diluted, filtered, and recombined into C as described below over 2 days in January 2022, just before the start of the incubation, during which all samples were stored at 4 °C and in the dark when not processed. All tubes containing the freeze-dried retained fractions (fractions 1 to 3) and U were dissolved in Milli-Q (Millipore) water and pooled together for the tubes corresponding to fractions 1, 2, or 3. The tubes were sonicated at least 3 times for 15 min at ca. 25 °C to help the material dissolve. The retained fractions and U and O samples were then filtered with pre-combusted GF/F filters to remove aggregates that could not be dissolved or that were formed during storage for O. After this, C was made by pooling U and the retained fractions in the same proportion as for O (i.e., by pooling together 20 mL of the fractions previously diluted in 200 mL). At day 0 of the incubation, concentrated artificial lake water containing nutrients and other macro- and microconstituents was added to each sample (U, 1, 2, 3, O, and C) to reach the concentrations given in Bastviken et al.^32^ (Table 1; TP 3.4 μg L^–1^ and TN 71 μg L^–1^) and included 10 mg L^–1^ NaHCO_3_ and KHCO_3_ to act as a buffer. In addition, a microbial inoculum from each respective site was added to each sample to constitute 2% of the total volume.^13,33^ All samples were further diluted with Milli-Q to reach an initial DOC concentration of ca. 10 mg L^–1^ (9.9 to 11.2 mg L^–1^), except for fraction 1 from the clearwater lake, which was diluted to 5.6 mg L^–1^ because of a lack of material. Note that the study design involved isolating DOM and separating it into polarity fractions, then redissolving the material into a standardized artificial lake water (common to all sites), and inoculating the samples with native bacteria from each site. Due to this approach, not everything about the water chemistry and biological community can be matched to in situ conditions. For example, all samples had a pH between 5.0 and 7.0 except two samples of the eutrophic site (pH = 2.7 and 3.2 for samples U and O, respectively), for which the low pH likely partially hindered degradation (Text S1). This pH effect did not alter the overall results and conclusions of the study since it only concerned two samples in one site, and the DOC loss of the most hydrophilic fraction was nevertheless higher than that of the hydrophobic fractions in this site (Text S1).

**Table 1:** Parameters of the Reactivity Continuum Model of Remaining DOC over Time and Predicted DOC Loss^a^

site (*R*^2^)	sample	*a*	*v*	*k*_0_	modeled DOC loss at 150 days (%)
clearwater lake	U	0.4 ± 0.1	0.048 ± 0.002	0.121	25
(0.99)	1	2.3 ± 0.5	0.031 ± 0.002	0.014	12
	2	5.1 ± 1.7	0.023 ± 0.003	0.004	7
	3	9.1 ± 3.7	0.023 ± 0.004	0.002	6
	C	0.4 ± 0.1	0.028 ± 0.001	0.071	15
	M	0.4 ± 0.1	0.031 ± 0.002	0.072	17
eutrophic lake	U	1.8 ± 0.3	0.036 ± 0.002	0.020	15
(0.98)	1	6.5 ± 0.8	0.069 ± 0.003	0.011	20
	2*	17.7 ± 3	0.069 ± 0.006	0.004	14
	3	20.4 ± 4.4	0.057 ± 0.006	0.003	11
	C	5.5 ± 0.9	0.049 ± 0.003	0.009	15
	M*	10.2 ± 2.5	0.053 ± 0.006	0.005	14
humic stream	U^†^	13.6 ± 2.4	0.068 ± 0.005	0.005	16
(0.97)	1	10.6 ± 2.4	0.068 ± 0.007	0.006	17
	2^§^	33.8 ± 7	0.095 ± 0.011	0.003	15
	3	29.2 ± 8.1	0.066 ± 0.01	0.002	11
	C^§^	45 ± 9.7	0.113 ± 0.015	0.003	15
	M^†^	7.6 ± 2.7	0.051 ± 0.008	0.007	14

a Samples: U unretained, most hydrophilic fraction; 1–3 retained fractions of increasing hydrophobicity; C and M experimental and theoretical remaining DOC of all fractions recombined in their original abundances. The model parameters that are not statistically different for the samples within each site share the same symbols (*†§). Fractions with no sign differ statistically from all other fractions within the site. *a* and *v* are the parameters given by the reactivity continuum model (given ± SE), *a* (rate parameter) relates more to the initial reactivity, and *v* (shape parameter) to the part where the curve levels off. A low *a* and a high *v* indicate a higher reactivity of DOC. *k*_0_, the initial apparent decay coefficient, was calculated as *v*/*a* as an indicator of DOC’s overall reactivity, it increases with the reactivity of DOC. For each site, the *R*^2^ of the model is given by the regression between measured and modeled data.

### DOC and pH Measurements

Each fraction as well as C and O samples were separated in 30 headspace-free vials at the start of the experiment for the DOC analysis of 2 replicate samples at 15 different time points (540 tubes in total). The samples were incubated in the dark at 20 °C. The initial O_2_ concentration was ca. 9 mg L^–1^. This was enough to maintain oxic conditions over the whole experiment, considering an initial DOC concentration of 10 mg L^–1^ and a maximum DOC loss of ca. 25%. DOC was measured with the TOC analyzer at days 0, 2, 5, 9, 14, weekly, and then bi-weekly over 150 days. pH was measured at the start and at the end of the incubation experiment in a 40 mL vial. In addition, 40 mL vials were prepared for MS analysis (two vials extracted and analyzed in duplicate each at the start and at the end of the experiment) and absorbance and fluorescence (two vials analyzed in triplicates at the start and at the end of the experiment).

### Solid-Phase Extraction

SPE was performed with 100 mg Bond Elut PPL cartridges (Agilent Technologies) within 10 days of the start of the incubation and within 2 days of the end (day 145). The cartridges were rinsed with methanol (hypergrade for LC–MS, Supelco), soaked in methanol for at least 2 h, and then rinsed with 0.1% formic acid. The samples (40 mL, duplicates) were acidified to pH ≈ 2 with 6 M high purity HCl (Suprapure, VWR; as 50% in Milli-Q, 2 mL L^–1^) and allowed to drip through the cartridges by gravity. The cartridges were flushed with 3 mL of 0.1% formic acid to remove salts and then dried using N_2_. The samples were eluted with 2 mL of methanol into pre-combusted 2 mL amber vials and stored at −20 °C until analysis.

To quantify how much DOC was recovered after SPE extraction, part of the SPE extracts (ca. 0.7 mL of MeOH) was dried down in a water bath, redissolved in Milli-Q water, sonicated for 15 min, and analyzed with the Sievers M9 TOC analyzer, after which the extraction efficiency was calculated.

### Characterization by MS

MS was performed on the other part of the SPE extracts. DOM samples were analyzed after separation on a size-exclusion chromatography column, and the material was simultaneously detected by ESI-MS and a charged aerosol detector (CAD). The separation did not reveal important changes to molecular weight distribution in SPE–DOM before and after incubation (Figures S2 and S3) and was not considered further in this study. 1 mL of SPE extracts were dried in a vacuum centrifuge and redissolved in 5% CH_3_CN (LCMS grade, Supelco, 200 μL). 30 μL of each sample was injected in a liquid chromatography method (Agilent 1100), which used 1 mL/min isocratic flow of 25 mM ammonium acetate in 20% MeOH as mobile phase on a size exclusion column (Tosoh TSK Gel G3000SW 300 × 7.5 mm, 10 μm pore size). Eluent was split and directed to a CAD to measure material abundance and a heated electrospray ionization mass spectrometer (LTQ-Velos Orbitrap, Thermo Fisher) operating in negative mode to measure the mass spectrum at approximately 1 transient per second. In this study, all transients were averaged together into a single peak list. All .raw and .mzXML files are available on the MassIVE data repository (MSV000092772).

One analytical blank and two PPL extraction blanks were analyzed. Peaks detected in samples that were less than 5× the intensity of the average blank were removed from consideration. SRFA was analyzed at five different concentrations in order to allow a comparison of intensities and abundance (from the CAD) of the reference material and the samples.

Potential doubly charged interferences were removed,^34^ along with spectral noise, and then formulas were assigned to the remaining peak list after internal calibration, first to mass 369.11911, and then to a series of masses that are common to all DOM samples. Combinations of C (4–50), H (4–100), O (2–40), N (0–2), and S (0–1) were allowed, along with up to one ^13^C. Allowed formulas had to be in the mass range 150–800, H/C 0.3–2.2, O/C < 1, double-bond equivalence minus oxygen 10 to −10, and could contain no more than one of the elements/isotopes N, S, and ^13^C.

Assigned sample peak lists were normalized and a Bray–Curtis dissimilarity matrix was calculated, which formed the basis of a principal coordinate analysis (PCoA). Finally, the sample-wise normalized intensity of each molecular formula was analyzed for correlation with sample position on principal coordinates 1 and 2 using Pearson’s rho to determine how the intensity of individual molecular formulas co-varied with overall molecular composition and sample dissimilarity. The full MATLAB code used for assignment, distance matrix, and PCoA and covariance testing is available in the Supporting Information.

### Characterization by Spectroscopic Techniques

UV–vis absorbance spectra (250 to 600 nm) were measured in a 1 cm quartz cuvette using a Lambda35 UV–vis spectrometer (PerkinElmer Lambda 25, PerkinElmer, Waltham, USA). Fluorescence scans were obtained using a FluoroMax-4 Spectrofluorometer (FluoroMax-4, Jobin Yvon, Horiba, Kyoto, Japan), with excitation-emission matrices (EEMs) from excitation wavelengths 250 to 445 nm with 5 nm increments and emission wavelengths 300 to 600 nm with 4 nm increments. A Milli-Q water blank run on the same day was used to correct the spectra; instrument biases and inner filter effects were corrected, and the spectra were normalized to Raman units^35,36^ using the FDOMcorr toolbox^37^ for MATLAB (The MathWorks, Inc., Natick, MA). The main DOM fluorescence components that varied throughout the data set were identified using PARAFAC.^38^ The analysis was conducted on a set of 114 samples (3 sites, 6 samples per site, 2 time points, triplicates, plus 6 EEMs from the pH test) using the drEEM toolbox for MATLAB (Mathworks, Inc., Natick, MA) following Murphy et al.^39^ Primary and secondary Rayleigh and Raman scattering were removed and smoothed over, and the data was normalized to the total fluorescence intensity of each sample. Nonnegativity constraints were applied to all modes (excitation, emission, and sample). The appropriate number of components was identified considering the effect of adding more components on the model fit (expressed as the sum of square errors), by visual inspection of the residuals and random initialization with 10 iterations with a convergence criterion of 1 × 10^–8^ to find a stable model. The model was validated using random split-half analysis by splitting the data set into three subsets. The model is uploaded and will be shared publicly upon publication in the OpenFluor database (URL:https://openfluor.lablicate.com/).

### Statistical Analyses

The fraction of remaining DOC at time *t* (DOC_*t*_/DOC_0_, unitless) was described using the reactivity continuum model that has previously been used in several inland water studies (e.g. refs (12)(13), and (40))
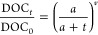
1*a* (days) is a rate parameter; it is the average lifetime of the more reactive DOC components. *v* (unitless) is a shape parameter and it relates to the preponderance of refractory compounds; a low *v* suggests the prevalence of refractory compounds.^12,41^*k*_0_ (day −1), the initial apparent decay coefficient, was calculated as *v*/*a* as an indicator of DOC overall reactivity.

A theoretical remaining DOC, denoted “M” in the rest of the article, was calculated as the sum of the remaining DOC (DOC_*t*_/DOC_0_, unitless) of the separated fractions (U, 1, 2, and 3), multiplied by the relative abundance of the fractions in the original sample.

The remaining DOC was modeled for all samples (U, 1, 2, 3, C, M, and O) and for each site (humic stream, eutrophic, and clearwater lakes) using a nonlinear model with fraction as a factor (gnls function; package nlme^42^). For the first retained fraction of the humic site, there was high variability of replicates from day 90, resulting in poor model performance. We consequently only included the remaining DOC until this day. The quality of the models was assessed by checking residuals and by plotting measured values against modeled values. The significance of the fixed effects on the model parameters was tested with ANOVA. In addition, we tested if the model parameters (*a* and *v*) significantly differed between the different samples within each site by comparing models with different sets of parameters with ANOVA.^43^ More specifically, this was done by testing if sharing both *a* and *v* for different samples decreased the model performance.

The correlation between the hydrophobicity of the fractions (U, 1, 2, and 3) and *k*_0_ was tested with a Spearman correlation. The proportion of the strong mobile phase (CH_3_CN) relates to the affinity of DOM for the hydrophobic stationary phase (i.e., the C_18_ column), which was used as a proxy of DOM’s hydrophobicity. The hydrophobicity of the fraction was consequently assessed by the weighted average proportion of CH_3_CN that was used to elute the fraction and was set to 0 for the unretained most hydrophilic fraction.

## Results and Discussion

### Consistent Highest Biodegradability of Hydrophilic Fractions

**Figure 1:**
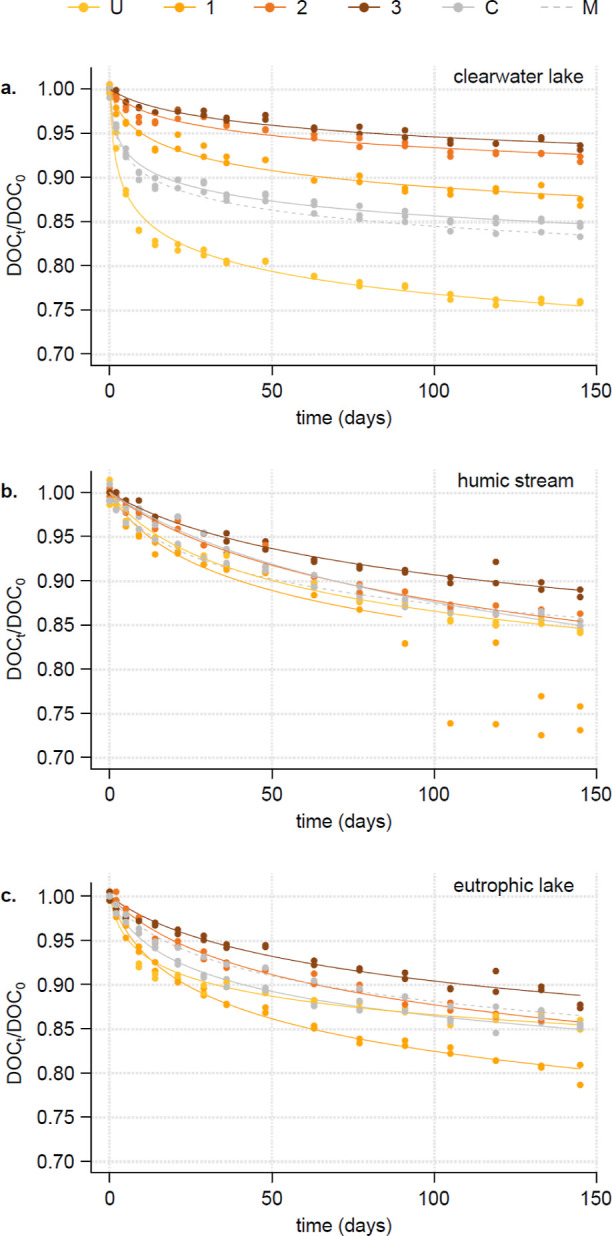
Measured (dots) fraction of remaining DOC (DOC_*t*_/DOC_0_, unitless) over time and reactivity continuum model (lines). U, 1, 2, and 3 are the DOM fractions of increasing hydrophobicity, U is the unretained and most hydrophilic fraction, and 3 is the most hydrophobic fraction. C is the recombination of U, 1, 2, and 3 in their original abundances. M is the theoretical remaining DOC of the recombined fractions, calculated assuming an additive effect.

**Figure 2:**
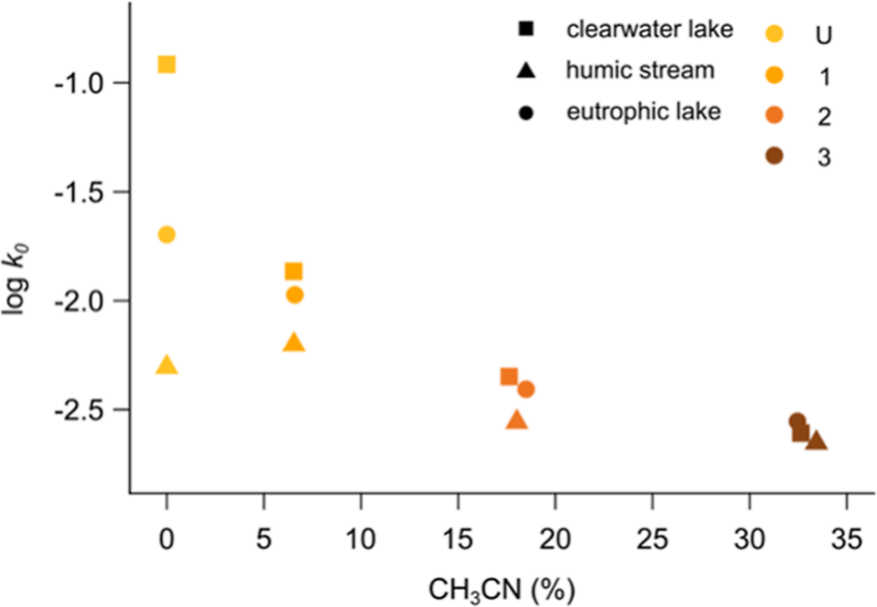
Correlation between the hydrophobicity of the different fractions (U, 1, 2, and 3), assessed by the weighted average proportion of the strong mobile phase used on the C_18_ column (CH_3_CN) to elute the fraction, and the initial decay constant (log *k*_0_). The hydrophobicity of the fractions increases with the eluent concentration; the concentration of the eluent was set to 0 for the most hydrophilic fraction, which was not retained by the column. The Spearman correlation coefficient was *r* = −0.915 (*p*-value = 2.9 × 10^–5^).

When DOM was separated into fractions of different hydrophobicity, differences in biodegradability emerged that were common for all sites. In all three sites, the most hydrophilic fractions (U and 1) were the most labile (*k* ≥ 0.005, 12–25% DOC loss at day 150; Table 1), and the most hydrophobic fractions (2 and 3) were the most refractory (*k* < 0.005, 6–15% DOC loss at day 150; Table 1 and Figure 1), supporting our hypothesis. There was however an overlap in biodegradability between the different fractions since all fractions comprised biodegradable DOC that was lost quickly in the first days of the incubation and more refractory DOC that remained at the end of the incubation. Nevertheless, a strong average effect was observed, evidenced by a strong and negative correlation between the initial constant decay and the hydrophobicity of the different fractions (Figure 2). This result, consistent for DOM obtained from three substantially different water bodies, reveals that hydrophobicity significantly contributes to the DOM reactivity continuum, with hydrophilic species being the most biodegradable.

**Figure 3:**
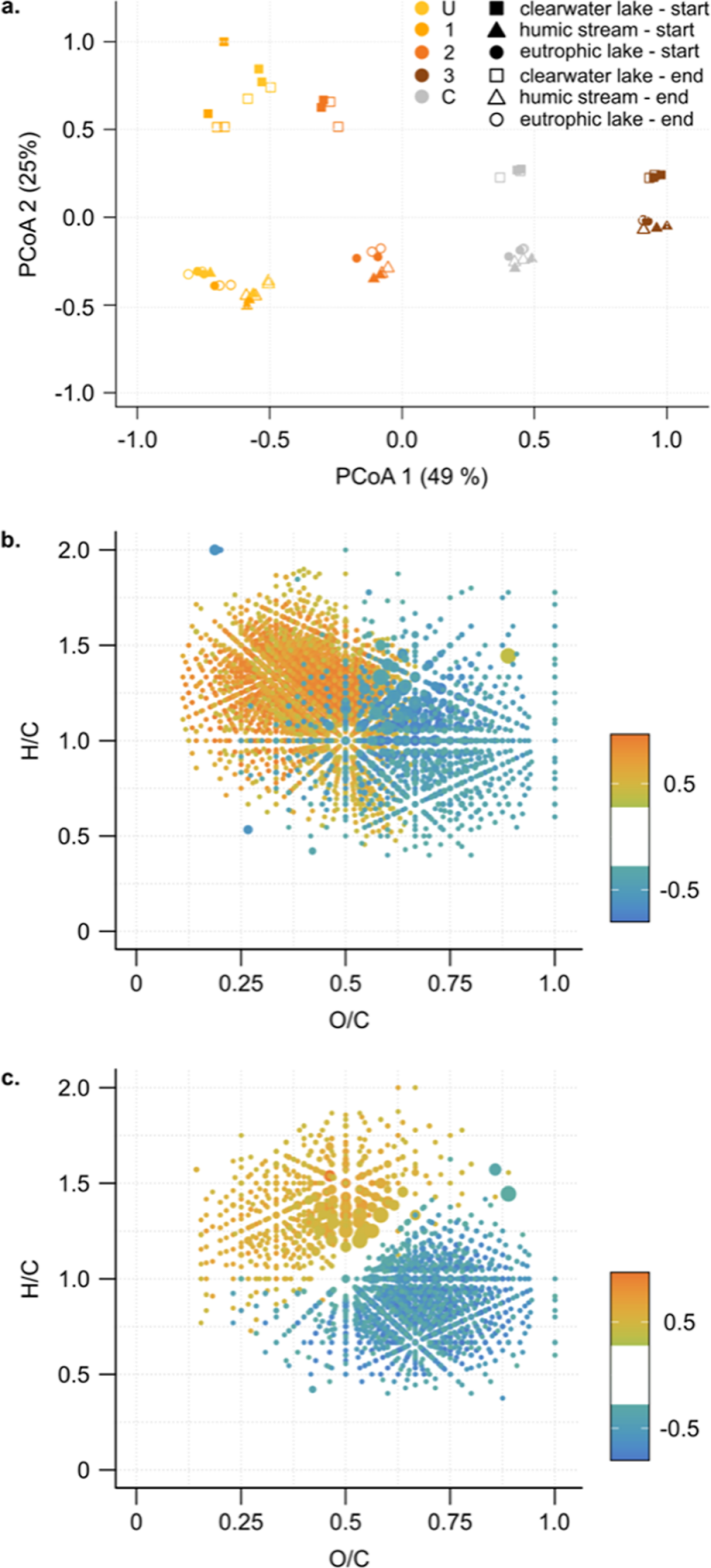
Differences in DOM composition between the samples (U, 1, 2, 3, and C) of the three sites and before/after incubation, as assessed by high-resolution MS. PCoA plot of the different samples in the three sites before and after incubation (a) and correlations between the first (b) and second (c) PCoA axes and the abundance of the individual molecular formulae (*n* = 3695 for PCoA1 and *n* = 1772 for PCoA2). In (b,c) the color scale indicates the significant Spearman correlations (|*r*| > 0.334 for *p*-value < 0.01 and *n* = 59 samples), and each molecular formula is represented by one dot according to its H/C vs O/C ratio.

We expected higher biodegradability for hydrophilic fractions because known labile biomolecular classes are hydrophilic (e.g., sugars and amino acids).^11,21^ It is, however, uncertain if the intrinsic character of hydrophilicity generally results in higher biodegradability. One reason for the higher biodegradability of hydrophilic molecules could be that chemical functional groups on which most biodegradation reactions are based (e.g., hydrolysis, oxidation) are often polar (e.g., O-containing functional groups). Indeed, in our study, the more hydrophilic fractions correlated with a higher abundance of high O/C compounds (Figure 3b, Text S2). Additionally, more hydrophobic DOM fractions could be relatively less biodegradable because hydrophobic species aggregate to decrease their extent of surface contact with water and, thus, indirectly, their accessibility to microorganisms.^30^

### Most Hydrophilic Compounds Are Outside of the SPE–ESI-MS Analytical Window

**Figure 4:**
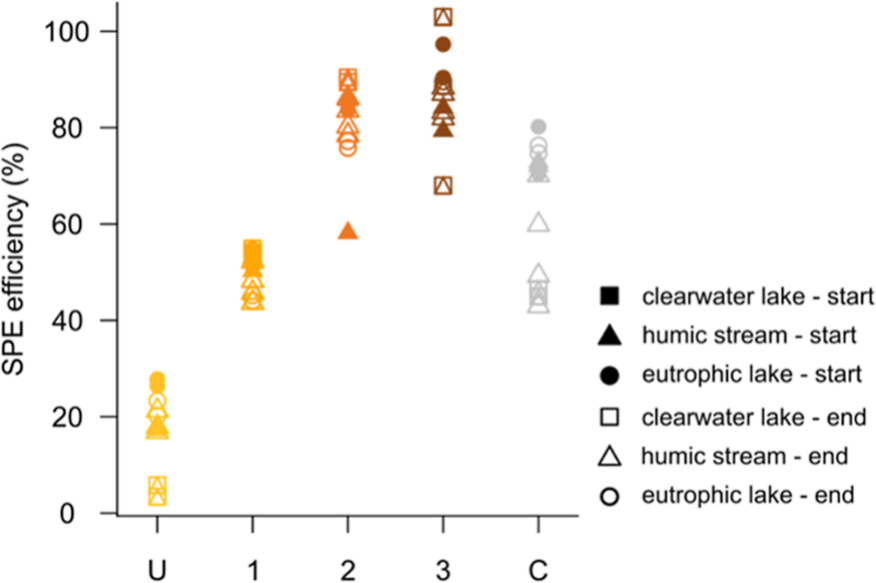
Percentage of DOC recovery after SPE efficiency in (%) for each sample “U” unretained and most hydrophilic fraction, 1–3 retained fractions of increasing hydrophobicity, and C all fractions recombined in their original abundances.

Our findings show that hydrophilic species are on average the most biodegradable, but such species are generally lost during SPE prior to MS analysis. The DOC percentage that was recovered after SPE and analyzed by MS (but not necessarily detected^44^) was low for the most hydrophilic fraction (3–28% for fraction U all sites combined, median 18%) but substantially higher for the most hydrophobic fractions (58–103% for fractions 2 and 3, median 85%, Figure 4). The percentage of DOC recovery after SPE extraction of bulk environmental water samples is generally around 60–70%,^7,45^ close to our recombined samples (43–80%, median 70% for sample C, Figure 4). SPE on hydrophobic sorbent retains hydrophobic compounds and excludes the most hydrophilic compounds.^28,29^ It is generally a necessity for MS approaches to use SPE to concentrate DOM and remove salts as a preliminary step (with a PPL cartridge, e.g.^7,24−26^), although a few studies have managed to analyze samples from freshwater environments without pre-concentration on PPL.^19,27,46^ The variability in DOC recovery between the fractions in this study was expected because these fractions were previously already separated using a non-polar stationary phase (C_18_). It also confirms that a significant part of the hydrophilic DOM is lost during SPE.^47^ Additionally, the comparison of ESI mass spectra before and after SPE shows little difference in the spectral results, indicating that not just extraction but also ionization and transfer to the gas phase in electrospray are inefficient for hydrophilic species.^28^ MS is consequently sub-optimal for investigating bulk DOM and its biodegradability. SPE is highly important prior to ESI-MS analysis, and cannot be removed as a preparation step—indeed, fewer molecular formulas may be assigned in this case due to lower sample concentrations and competition for electrospray from salts.^48^ Studies that aim to characterize biodegradable DOM may require alternative preparative and analytical techniques, for example, focusing on sugar and protein compound classes after ultrafiltration, as this has been successful previously,^49,50^ and such efforts could be combined with PPL ESI-MS approaches for more complete sample coverage. Some compounds, which are most similar to inorganic salts (i.e., both small and hydrophilic), may remain challenging to include in high-resolution analytical techniques and may require targeted methods.

Consistent with the limited ability of the MS analysis to explain changes in DOM biodegradability, we also found a limited measurable change in MS composition before and after incubation (Figure 3a). Differences in composition between fractions of different hydrophobicity and between sites were generally much more important than those before and after incubation. The dissimilarity in MS spectra before and after incubation, as quantified with the Bray–Curtis metric was on average 14 ± 14% (all fractions and all sites combined) and was the highest (28 ± 20%) for the most hydrophilic fraction (fraction U all sites combined). This was low considering that incubation replicates had an 11 ± 13% dissimilarity. Our quantitative approach, based on DOC concentration, to describing bulk DOM biodegradability in relation to hydrophobicity is thus more broadly inclusive than the MS peak abundance approach. There has been substantial recent progress in the understanding of how the reactivity of DOM is related to its composition. However, this progress builds heavily on MS analysis,^7,19,25^ which suffers from loss of material during extraction as well as biases due to incomplete ionization. Future studies should investigate if the current knowledge still holds when assessing bulk DOM reactivity.

### Challenging the Established Relationships between DOM Biodegradability and the H/C Ratio

Despite the general low extraction efficiency and ionization coverage of the biodegradable DOM, we were able to see a consistent change in DOM composition before and after bio-incubation (Figure S4). Inspection of the mass of the molecular formulas lost revealed that the higher molecular mass compounds, especially those with comparatively fewer double bond equivalents, were more labile (Figure S5), corresponding well with the “size-reactivity continuum” theory.^15^ The DOM composition changes were reproducible across the three sites. In the most hydrophobic fraction, lipid-like compounds with H/C > 1.5 and O/C < 0.5 were preferentially removed (fraction 3, Figure S4). In the other fractions, the most oxygenated species (O/C > 0.6) were the most prone to removal (fractions U, 1, and 2, Figure S4), as also found by other studies.^20,51,52^ This result complicates the prevailing concept that DOM degradability or persistence is mainly driven by aromaticity or H/C ratio.^7,12,19,26,53−55^

### Convergence of DOM Composition and Biodegradability at Higher Hydrophobicity

Both the DOM biodegradability and measured composition of the three lakes converged with increasing hydrophobicity (Table 1, Figure 3a). Accordingly, the biodegradability of the most hydrophilic fractions varied greatly between sites; for example, the biodegradability of the hydrophilic fraction of the clearwater lake was up to 20 times higher than that of the other sites (*k*_0_ of fraction U, Table 1). Conversely, the biodegradability of the most hydrophobic fractions was contained in a narrow range (0.002 < *k*_0_ < 0.004, fractions 2 and 3, Table 1). In addition, within the applied analytical window, the DOM composition between the clearwater lake and the other sites became more similar with increasing hydrophobicity (PCoA2, Figure 3a). DOM in the eutrophic and humic sites was generally more enriched in oxygen-rich, aromatic compounds, often referred to as “tannins” (Figure 3c), which are generally hydrophilic, than in the clearwater lake. However, as hydrophobicity increased, Bray–Curtis dissimilarity between the sites decreased, showing that compounds in hydrophobic DOM fractions are more similar than in hydrophilic fractions (PCoA2, Figure 3a). Since the most hydrophobic fractions are also the most stable, this result suggests that recalcitrant species across landscapes have compositional similarities. Our finding may explain the convergence in the composition of stable DOM that persists in waters with a long retention time (e.g., “island of stability” of DOM found in seawater^56−58^).

### No Consistent Interactive Effect on Biodegradability When All Fractions Are Degraded Together

The three sites showed different patterns when all fractions were combined and incubated together. The DOC loss after 150 days in incubations where all fractions were recombined (C) was similar to what was theoretically expected (M), between 14 and 17% for C and M samples (Table 1). However, for all three sites, the reactivity continuum had a different shape from what is theoretically expected without an interaction between fractions (i.e., no shared symbol between samples C and M in Table 1), although the difference was sometimes visibly low (clearwater lake, Figure 1). For one site, the recombined sample (C) had a significantly higher biodegradability, while for the two other sites, it had a lower biodegradability than expected (M) (Table 1). This suggests that there is no consistent synergistic or antagonistic effect when all fractions are degraded together. The mechanisms that would enhance or limit the degradation of different organic fractions are complex (described in detail in Sanches, Guenet, Marino, and Esteves^22^ and Bengtsson, Attermeyer, and Catalán^23^) and could be limited in aquatic ecosystems in comparison to soils, explaining why we did not find a consistent effect.^23^ Alternatively, interactive effects occurred within one or several of the broad fractions that we examined, i.e., the components of DOM that interacted were not resolved. In addition, the average difference in biodegradability between the fractions (Table 1) was possibly insufficient to result in a detectable and consistent effect across all three sites. Indeed, such interactive effects have been observed following the addition of single compounds (e.g., glucose) or in situ-produced DOM^59−61^ that are likely to have a higher biodegradability than the most hydrophilic fractions in our experiment.

## Data Availability

Mass spectrometry data are available at MassIVE archive MSV000092772.

## Supporting Information Available

Effects of sample preparation and pH on DOM biodegradability; DOM composition of fractions with different hydrophobicity; mass distribution of the fractions separated from three water samples according to their retention time in the C18 column; size-exclusion chromatograms of material eluting from the column and detected by CAD per site; size exclusion chromatograms of material eluting from the column and detected by CAD with fractions separated into individual plots; fraction by fraction analysis of compositional changes (H/C vs O/C ratio) detected by MS; fraction by fraction analysis of compositional changes (DBE vs *m*/*z*) detected by MS; measured (dots) fraction of remaining DOC (DOC_*t*_/DOC_0_, unitless) over time and reactivity continuum model (lines) including the original sample O; fraction of remaining DOC over time for the original sample of the eutrophic lake at different pH; intensity of the PARAFAC model components for each of the three sites; characteristics of the three sampling sites; parameters of the reactivity continuum model of remaining DOC over time; and predicted DOC loss for O and C samples (PDF)
