# Supplementary material for: Hydrophilic Species Are the Most Biodegradable Components of Freshwater Dissolved Organic Matter
Source: Environ Sci Technol. 2023 Aug 30;57(36):13463–72. doi: 10.1021/acs.est.3c02175 (PMC10501193; doi:10.1021/acs.est.3c02175)
Supplement: Supplementary file 1 — es3c02175_si_001.pdf [file es3c02175_si_001.pdf]

Supplementary information

Hydrophilic species are the most biodegradable components of freshwater dissolved organic matter

Charlotte Grasset[1,§], Marloes Groeneveld[1,§], Lars J. Tranvik[1], Luke P. Robertson[2], Jeffrey A. Hawkes[3]*

1. Department of Ecology and Genetics, Limnology, Uppsala University, Sweden
2. Department of Pharmaceutical Biosciences, Uppsala University, Sweden
3. Department of Chemistry, BMC, Uppsala University, Sweden
[§] Contributed equally
* Corresponding author: Jeffrey Hawkes, jeffrey.hawkes@kemi.uu.se

Number of pages: 12
Number of texts: 2
Number of tables: 2
Number of figures: 8

Text S1. Effects of sample preparation and pH on DOM bioreactivity

In the eutrophic and humic sites, the O (original sample) and C (U, 1, 2, 3 recombined to recreate the original sample) experiments did not match, with O being visibly less biodegradable than C (Fig S6) and having a lower predicted DOC loss (Table S2). In addition, k_0 was in average three times higher for C than for O samples for all systems (Table S2). This indicates some sample preparation effect after separation which overestimates the reactivity of these samples (i.e. U, 1, 2, 3 and C), but presumably not the relative differences between the samples. While most fractions had a pH around 6 at the start of the incubation, eutrophic lake samples U and O had a pH of 2.7 and 3.2, respectively. We tested if the low pH could explain the slower decomposition rate of the O sample in comparison to the C sample (initial pH=5.04). The increase in pH of the O sample, by adding 1M $NaHCO_3$, indeed increased decomposition rates for the eutrophic lake (Fig S7). However, for the humic and clearwater sites, the slower decomposition rate of the O sample in comparison to the C sample cannot be explained by a pH effect since all samples had a pH around 6 and could be explained by other differences in water chemistry after sample fractionation (e.g. related to freeze-drying or sonication steps).

Text S2. DOM composition of fractions with different hydrophobicity

According to our MS analysis, when the most hydrophilic species were excluded, there was a clear separation of the samples along the O:C axis (at O:C =0.5); compounds with low O:C ratios being more abundant in the more hydrophobic fractions (Fig 3). This suggests that more generally hydrophobic compounds tend to have a lower O:C ratio, likely because of the polarity of functional groups that contain O (e.g. hydroxyl or carboxylic acid groups).

The fluorescence analysis showed different results from the MS analysis, with a relatively higher content in humic-like compounds for hydrophobic fractions (usually being correlated to low H:C[1]) (Fig S8).This discrepancy between MS and fluorescence analyses can be explained by their different analytical windows. In addition to excluding the most hydrophilic compounds, aromatic and large compounds are not well ionized and thus detected by MS[2], while they are efficient to absorb light and thus well detected by fluorescence.

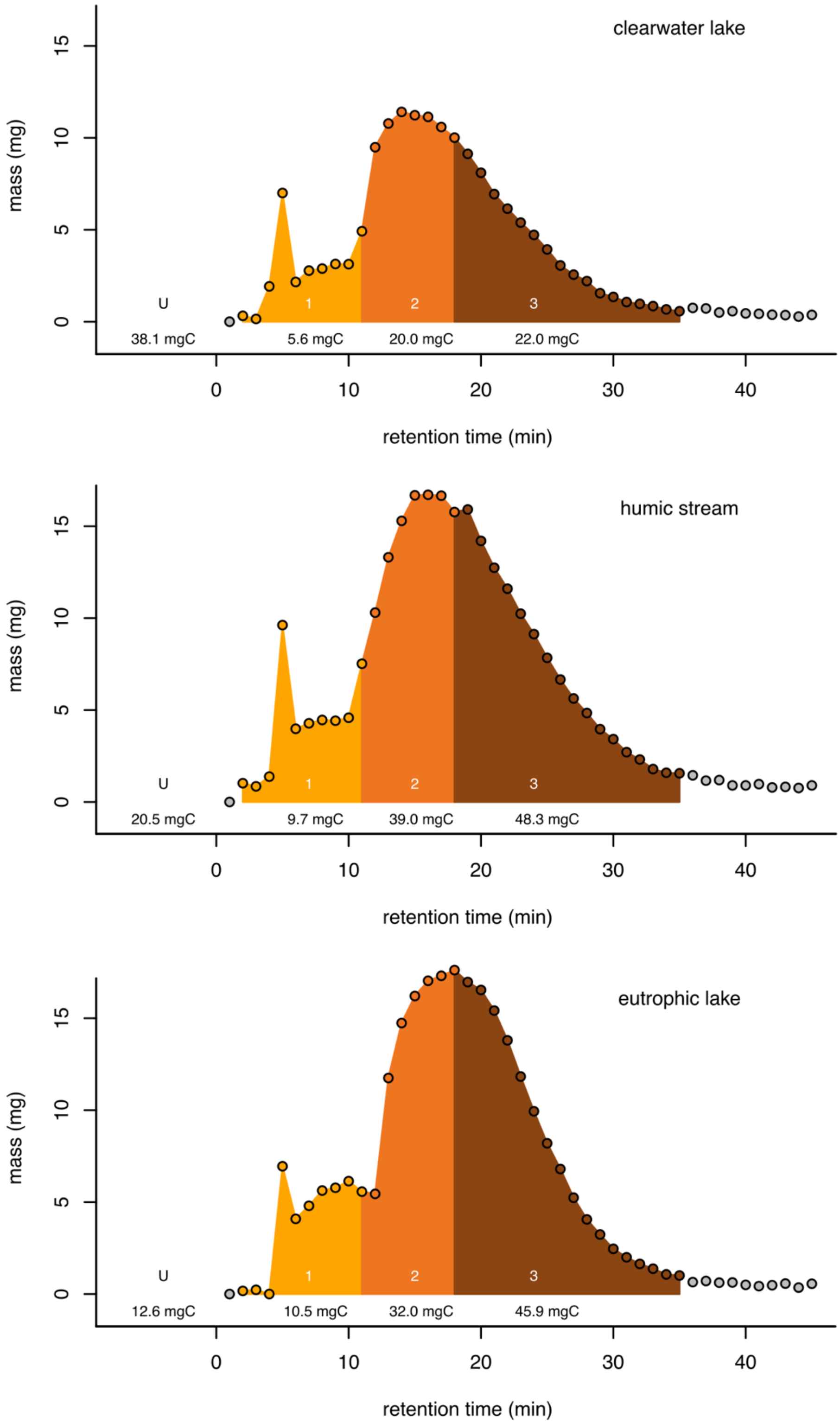

Figure S1. Mass distribution of the fractions separated from three water samples (clearwater and eutrophic lakes and humic stream) according to their retention time in the C_{18} column. Each dot is the weight of the material collected every min in the test tubes during the elution of the C_{18} column with CH_3CN to constitute the fractions '1', '2' and '3'. The weight of the unretained fraction ('U') is represented at time 0.

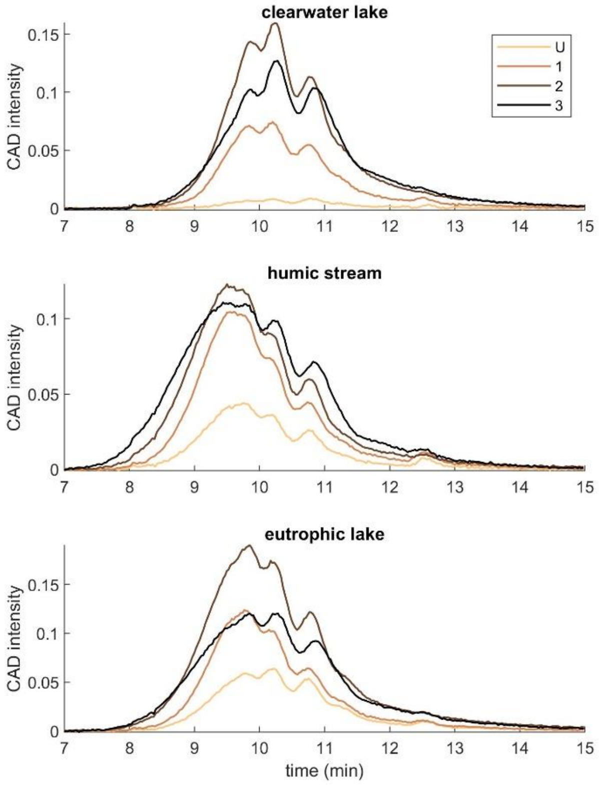

Figure S2: Size exclusion chromatograms of material eluting from the column and detected by charged aerosol detector. Earlier eluting material is higher molecular weight, and charge density also has an exclusion effect, making more charged compounds appear higher molecular weight. Only one replicate is shown for each fraction, from the beginning of the incubation. The humic stream, in particular, has some high molecular weight material in the more hydrophobic fraction.

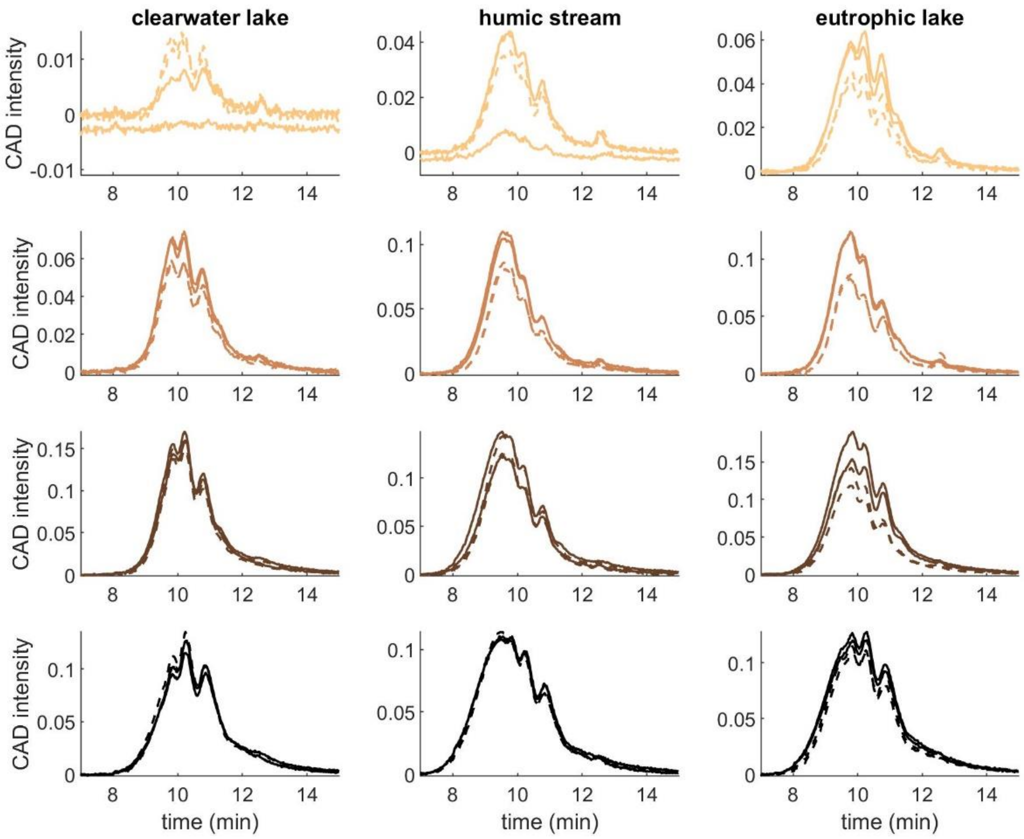

Figure S3: Size exclusion chromatograms of material eluting from the column and detected by charged aerosol detector. Here, the fractions are separated into individual plots, and both replicates are shown for the beginning (solid line) and end (dashed line) of the incubation. Generally, the end of the incubation had less material eluting (particularly for the more hydrophilic fractions, as expected). Note that this analysis was done on solid phase extracts, and so does not capture all DOM, and in particular does not reflect all of the lost DOC. Notably, the loss of DOM observed in some cases after incubation did not seem to affect the molecular weight distribution of DOM.

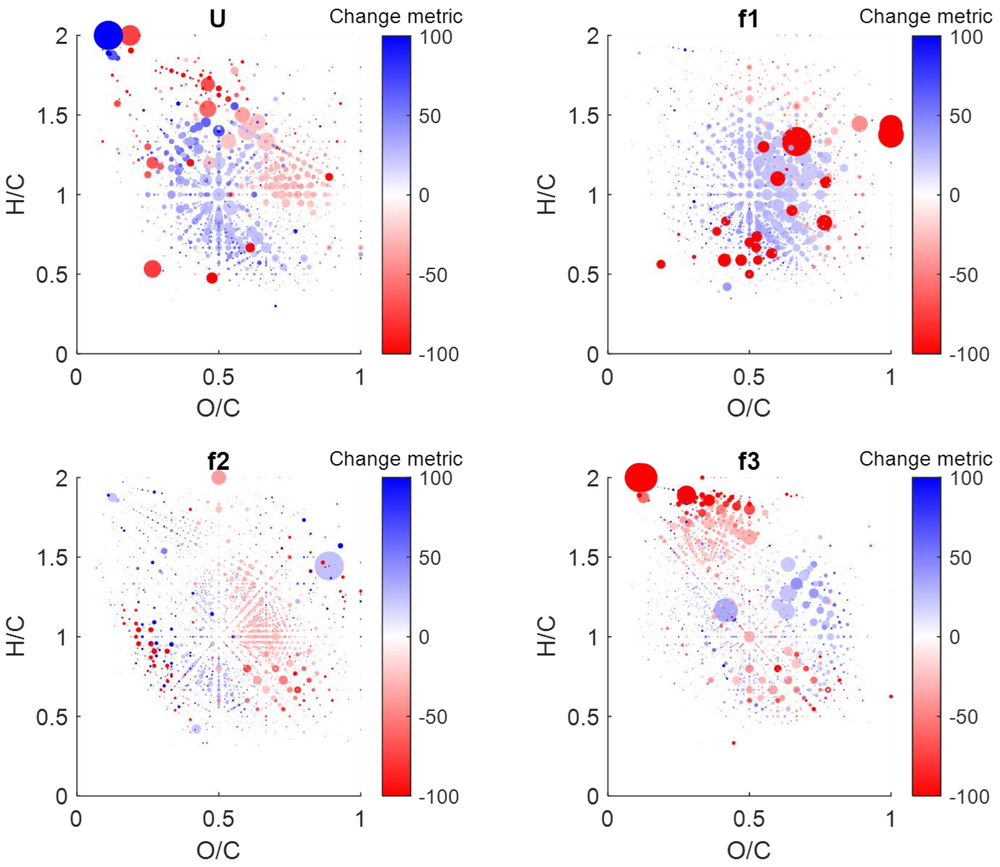

Figure S4. Fraction by fraction analysis of compositional changes detected by MS, all sites combined. Each molecular formula is represented by one dot according to its H:C vs. O:C ratio. The colour axis is calculated as (end-start)/(end+start)*100 and only changes > |20| are depicted. Blue is relatively enriched and red is relatively lost after incubation. The point size is the average relative intensity at the start. Before calculating the relative change, the two replicate spectra for each site at the start and at the end of the incubation were averaged.

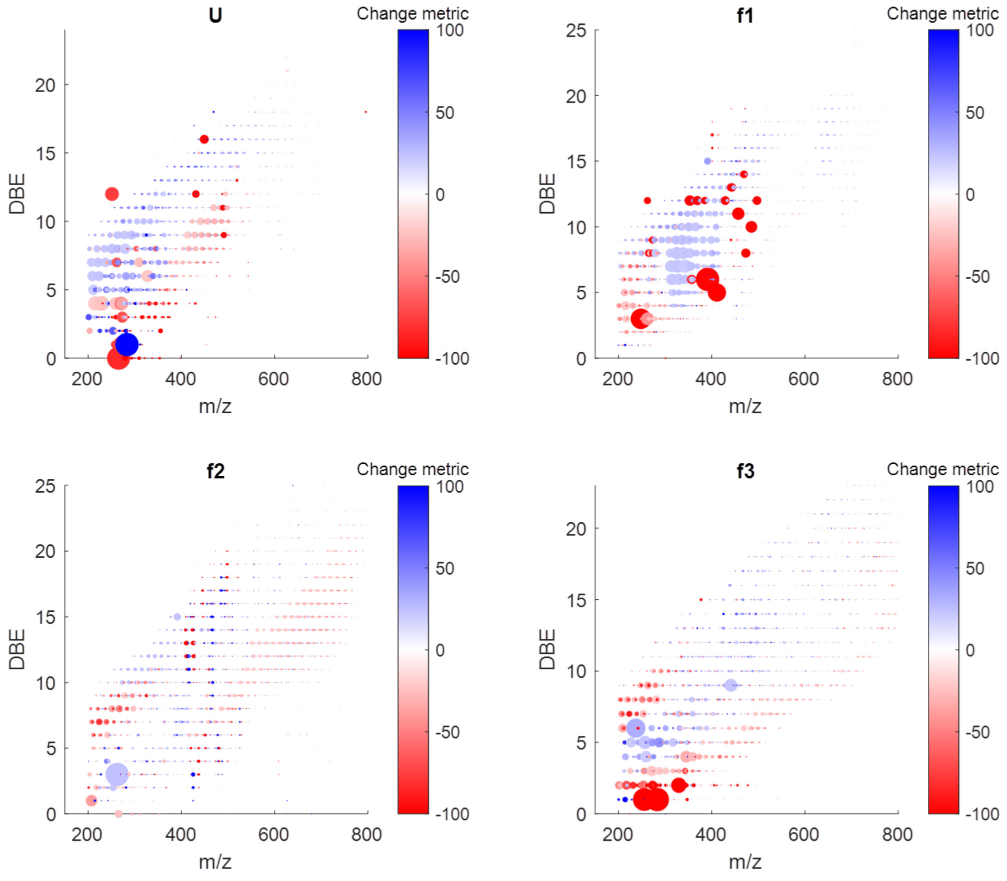

Figure S5. Fraction by fraction analysis of compositional changes detected by MS, all sites combined. Each molecular formula is represented by one dot according to its double bound equivalent (DBE, indicates the degree of unsaturation) vs. mass divided by charge number (m/z). The colour axis is calculated as (end-start)/(end+start)*100 and only changes > |20| are depicted. Blue is relatively enriched and red is relatively lost after incubation. The point size is the average relative intensity at the start. Before calculating the relative change, the two replicate spectra for each site at the start and at the end of the incubation were averaged.

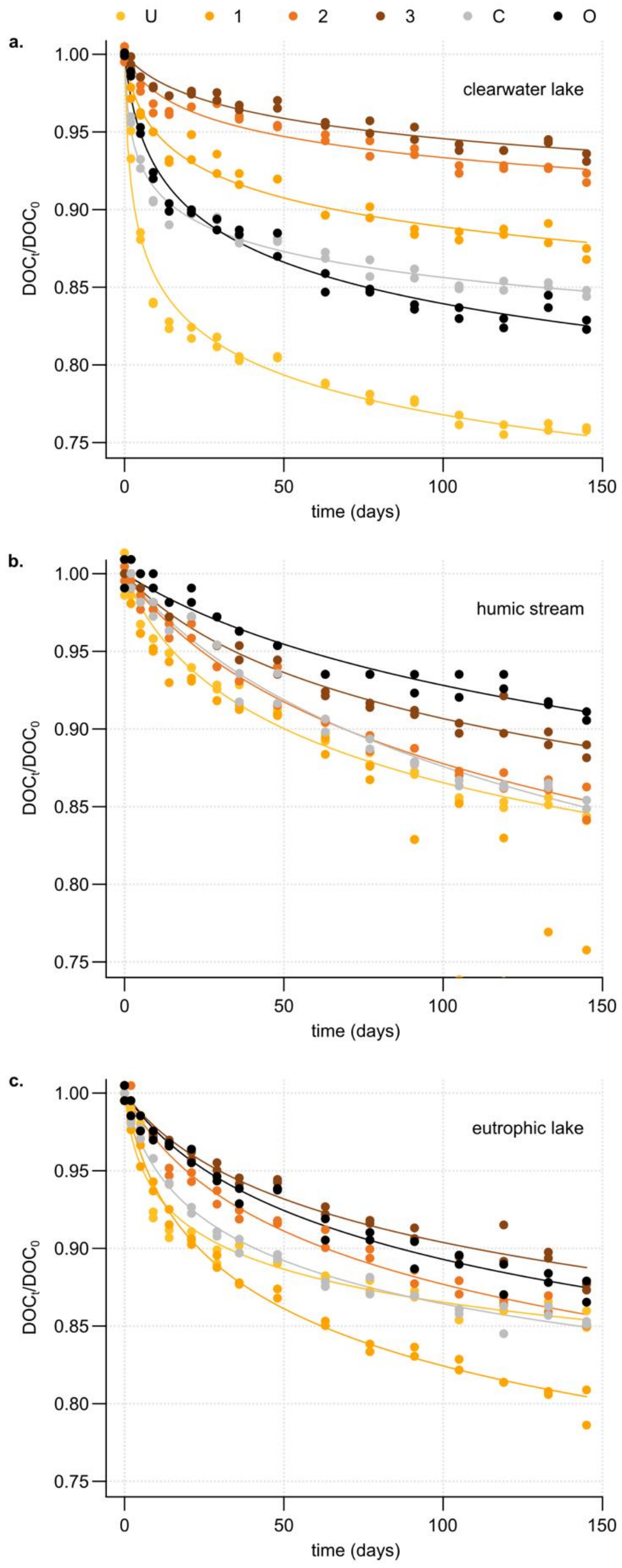

Figure S6. Measured (dots) fraction of remaining DOC (DOC_t/DOC_0, unitless) over time and reactivity continuum model (lines) including the original sample O. U, 1, 2 and 3 are the DOM fractions of increasing hydrophobicity, U being the most hydrophilic fraction and 3 being the most hydrophobic. O is the original sample that was fractionated and C is the recombination of U, 1, 2 and 3 in their original abundances.

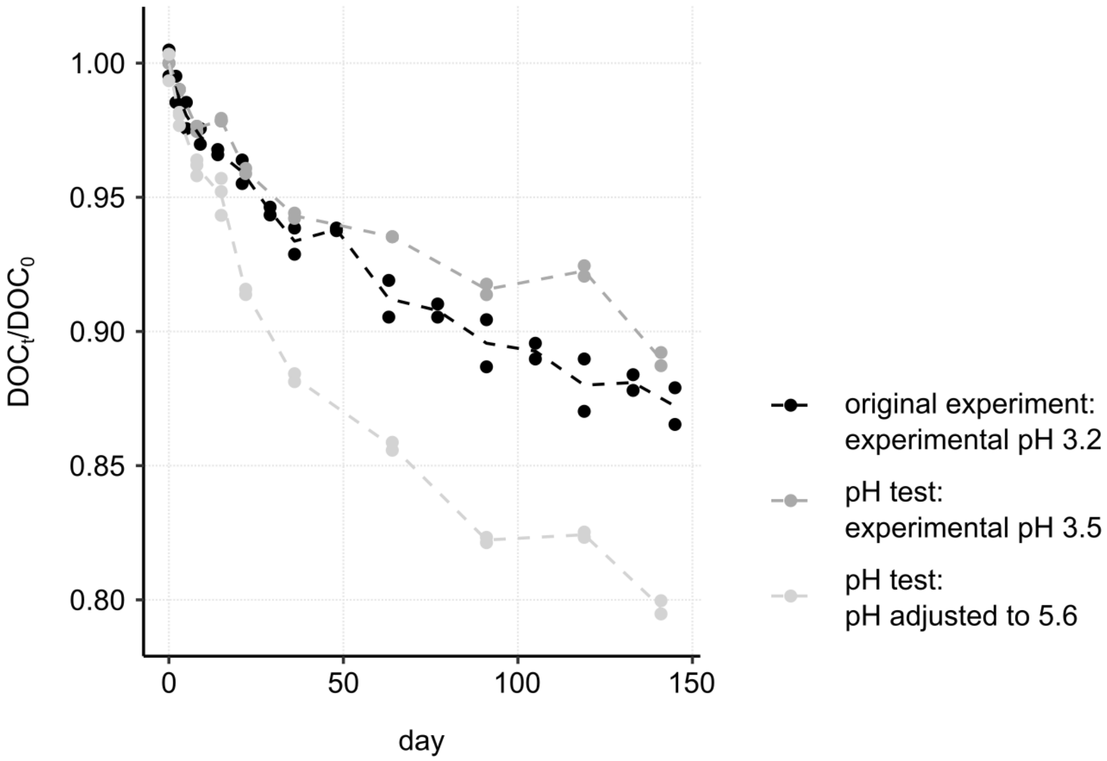

Figure S7. Fraction of remaining DOC over time for the original sample of the eutrophic lake at different pH. Remaining DOC is presented for three samples, the original sample used in the experiment presented in the manuscript (pH 3.2) and a new batch of the eutrophic lake original sample at its experimental pH (3.5) and adjusted pH (5.7). The pH was adjusted with 1M $NaHCO_3$ (250μL in 450 mL).

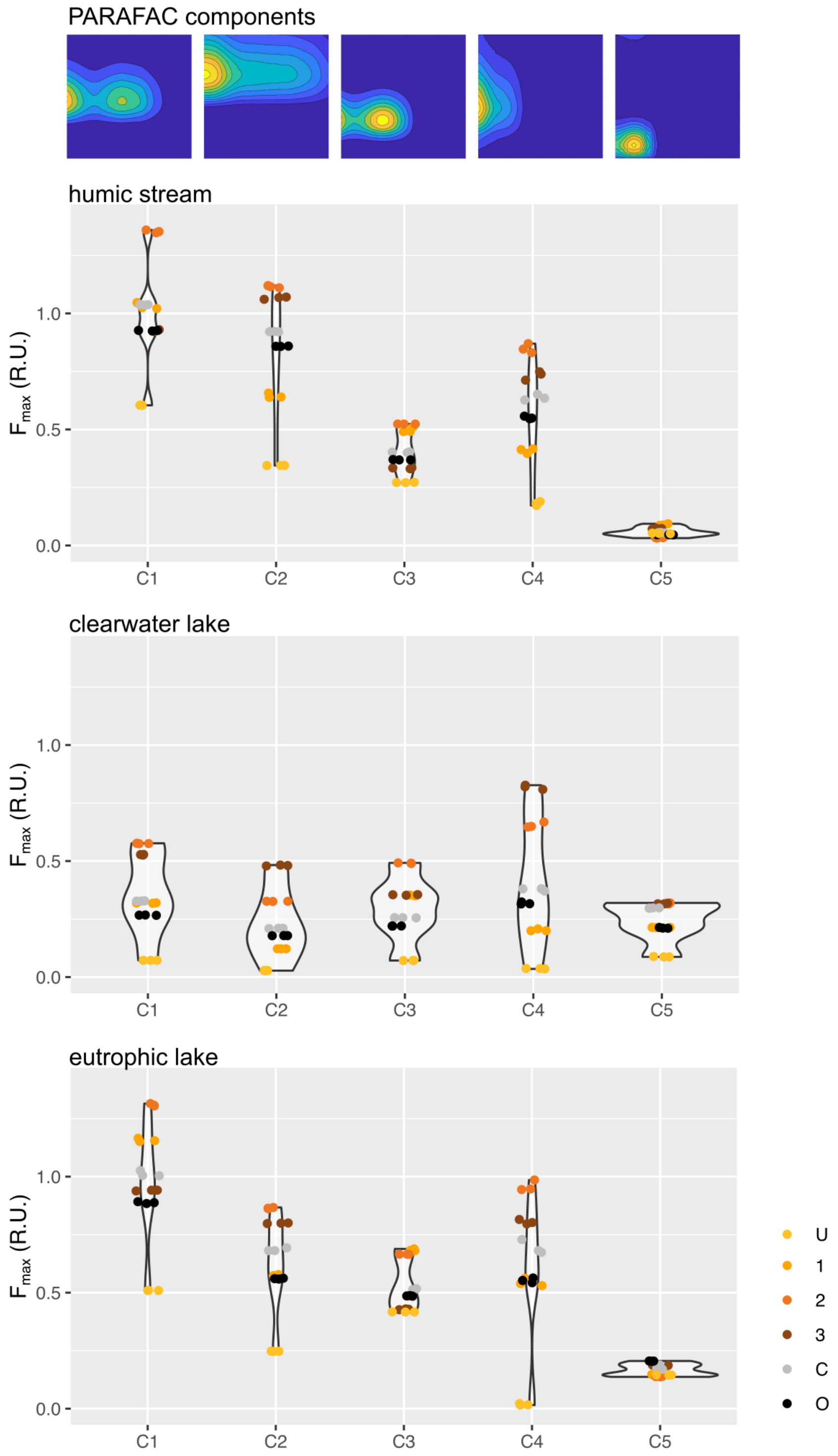

Figure S8. Intensity of the PARAFAC model components for each of the three sites. The components have their maximum at the following excitation/emission wavelengths: C1 = 250/436 nm, C2 = 250/504 nm, C3 = 315/392 nm, C4 = 250/436 nm, C5 = 280/332 nm. U, 1, 2 and 3 are the DOM fractions of increasing hydrophobicity, C is the recombination of U, 1, 2 and 3 in their original abundances and O is the original sample. The violin plot outlines illustrate the kernel probability density where the width outline represents the proportion of the data located there.

Table S1. Characteristics of the three sampling sites.

**Site**	**humic stream**	**clearwater lake**	**eutrophic lake**
**Name**	Fiby	Långsjön	Alstasjön
**Latitude (°N)**	59.91483	59.26733	59.74298
**Longitude (°E)**	17.34952	17.96565	17.265675
**DOC (mgL^{-1})**	46.4	6.0	23.2
**TP (µgL^{-1})**	34.1	65.3	297.8
**TN (mgL^{-1})**	1.26	0.52	2.9
**pH**	5.9	8.0	7.6
**Catchment area (km^2)***	19.2	28.5	42.9
**Catchment land use***			
forest (%)	79	43	41
wetland (%)	19	1	1
agriculture/fields (%)	0	42	55
lake (%)	2	9	3
other (%)	0	5	0

* Catchment data for the humic stream is given for the entire catchment of Fibysjön, the lake that the stream drains in to.

Table S2. Parameters of the reactivity continuum model of remaining DOC over time and predicted DOC loss for O and C samples.

Site	Sample	***a***	***v***	***k_0***	**modelled DOC loss at 150 days (%)**
clearwater lake	O	2.4 ± 0.4	0.047 ± 0.002	0.019	18
	C	0.4 ± 0.1	0.028 ± 0.001	0.071	15
eutrophic lake	O	21.5 ± 4.2	0.065 ± 0.006	0.003	13
	C	5.5 ± 0.9	0.049 ± 0.003	0.009	15
humic stream	O	55.9 ± 21.6	0.073 ± 0.018	0.001	9
	C	45 ± 9.7	0.113 ± 0.015	0.003	15

The model parameters are statistically different for the samples within a same site.

References

1 Kellerman, A. M., Kothawala, D., Dittmar, T. & Tranvik, L. J. Persistence of dissolved organic matter in lakes related to its molecular characteristics. *Nature Geoscience* **8**, 454-457, doi:10.1038/ngeo2440 (2015).

2 Patriarca, C. *et al.* Investigating the Ionization of Dissolved Organic Matter by Electrospray. *Analytical Chemistry* **92**, 14210-14218, doi:10.1021/acs.analchem.0c03438 (2020).
